# Atrial Fibrillation in Patients With Cardiomyopathy: Prevalence and Clinical Outcomes From Real‐World Data

**DOI:** 10.1161/JAHA.121.021970

**Published:** 2021-11-15

**Authors:** Benjamin J. R. Buckley, Stephanie L. Harrison, Dhiraj Gupta, Elnara Fazio‐Eynullayeva, Paula Underhill, Gregory Y. H. Lip

**Affiliations:** ^1^ Liverpool Centre for Cardiovascular Science University of Liverpool and Liverpool Heart & Chest Hospital Liverpool UK; ^2^ Department of Cardiovascular and Metabolic Medicine Institute of Life Course and Medical Sciences, University of Liverpool Liverpool UK; ^3^ Liverpool Heart and Chest Hospital Liverpool UK; ^4^ TriNetX LLC. Cambridge MA; ^5^ TriNetX LLC. London UK; ^6^ Department of Clinical Medicine Aalborg University Aalborg Denmark

**Keywords:** atrial fibrillation, cardiomyopathy, comorbidity, MACE, preventive cardiology, secondary prevention, Atrial Fibrillation, Cardiomyopathy, Epidemiology, Secondary Prevention

## Abstract

**Background:**

Cardiomyopathy is a common cause of atrial fibrillation (AF) and may also present as a complication of AF. However, there is a scarcity of evidence of clinical outcomes for people with cardiomyopathy and concomittant AF. The aim of the present study was therefore to characterize the prevalence of AF in major subtypes of cardiomyopathy and investigate the impact on important clinical outcomes.

**Methods and Results:**

A retrospective cohort study was conducted using electronic medical records from a global federated health research network, with data primarily from the United States. The TriNetX network was searched on January 17, 2021, including records from 2002 to 2020, which included at least 1 year of follow‐up data. Patients were included based on a diagnosis of hypertrophic, dilated, or restrictive cardiomyopathy and concomitant AF. Patients with cardiomyopathy and AF were propensity‐score matched for age, sex, race, and comorbidities with patients who had a cardiomyopathy only. The outcomes were 1‐year mortality, hospitalization, incident heart failure, and incident stroke. Of 634 885 patients with cardiomyopathy, there were 14 675 (2.3%) patients with hypertrophic, 90 117 (7.0%) with restrictive, and 37 685 (5.9%) with dilated cardiomyopathy with concomitant AF. AF was associated with significantly higher odds of all‐cause mortality (odds ratio [95% CI]) for patients with hypertrophic (1.26 [1.13–1.40]) and dilated (1.36 [1.27–1.46]), but not restrictive (0.98 [0.94–1.02]), cardiomyopathy. Odds of hospitalization, incident heart failure, and incident stroke were significantly higher in all cardiomyopathy subtypes with concomitant AF. Among patients with AF, catheter ablation was associated with significantly lower odds of all‐cause mortality at 12 months across all cardiomyopathy subtypes.

**Conclusions:**

Findings of the present study suggest AF may be highly prevalent in patients with cardiomyopathy and associated with worsened prognosis. Subsequent research is needed to determine the usefulness of screening and multisdisciplinary treatment of AF in this population.

**Nonstandard Abbreviations and Acronyms**
CASTLE AF: Catheter Ablation Versus Standard Conventional Therapy in Patients With Left Ventricular Dysfunction and Atrial Fibrillation
EMR: electronic medical record
PSM: propensity score matching
RE‐LY: Randomized Evaluation of Long‐Term Anticoagulation Therapy
STROBE: Strengthening the Reporting of Observational Studies in Epidemiology

**Clinical Perspective**

**What Is New?**

- We investigated the prevalence and associated clinical outcomes of concomitant atrial fibrillation and cardiomyopathy subtypes.
- In this large retrospective cohort study, atrial fibrillation prevalence was 23.6%, 42.5%, and 44.4% in patients with hypertrophic, restrictive, and dilated cardiomyopathies, respectively.
- Concomitant atrial fibrillation was associated with increased odds of mortality in all but restrictive cardiomyopathy cohorts. Odds of hospitalization, incident heart failure, and incident stroke were significantly higher in all cardiomyopathy subtypes with concomittant atrial fibrillation.

**What Are the Clinical Implications?**

- In this large sample of patients with cardiomyopathy, atrial fibrillation is highly prevalent and associated with worsened prognosis.
- Subsequent prospective research is warranted to investigate the impact of screening and multidisciplinary treatment strategies.

Atrial fibrillation (AF) is the most common cardiac arrhythmia and is associated with various cardiovascular risk factors, which in turn contribute to the risk of AF‐related complications.^1^ In contemporary anticoagulated AF populations, the majority of deaths are related to causes other than stroke. For example, the RE‐LY (Randomized Evaluation of Long‐Term Anticoagulation Therapy) trial^2^ reported that progressive heart failure (HF) and sudden cardiac death accounted for 15% and 22% of deaths in patients with AF, respectively, whereas stroke accounted for 7%. Further studies have also reported a high risk of cardiovascular adverse events, aside from stroke and despite anticoagulation use, in patients with AF.^3^ These findings emphasize the need for a more holistic or integrated care approach to AF management to further reduce mortality in patients with AF.

Cardiomyopathies are myocardial disorders that are not secondary to coronary disease, hypertension, and congenital, valvular, or pericardial abnormalities. Four main subtypes of cardiomyopathy are hypertrophic, dilated, restrictive, and less commonly, arrhythmogenic right ventricular cardiomyopathy. Cardiomyopathy is a common cause of new onset AF and may also present as a sequela of AF. When presented together, patients suffer from worse symptoms and poorer prognosis.^4^ However, evidence‐based evaluation and management of this complex patient group is lacking.

The majority of previous real‐world data have typically comprised small samples, especially when investigating population subgroups and outcomes such as mortality. Also, although several studies have focused on AF with hypertrophic cardiomyopathy, there are limited published data on dilated and restrictive cardiomyopathies, especially with regard to the impact of AF on important clinical outcomes in these populations. To address this, we investigated the prevalence and clinical impact of AF in patients with cardiomyopathy across patient characteristics and different cardiomyopathy subtypes.

## Methods

### Data Availability Statement

To gain access to the data in the TriNetX research network, a request can be made to TriNetX (https://live.trinetx.com), but costs may be incurred, a data sharing agreement would be necessary, and no patient‐identifiable information can be obtained.

### Study Design and Participants

A retrospective observational study was conducted within TriNetX, a global federated health research network with access to electronic medical records (EMRs) from participating health care organizations including academic medical centers, specialty physician practices, and community hospitals covering ~69.8 million individuals, predominantly in the United States, from which we have previously published. More information on the database can be found online (https://trinetx.com/company‐overview/). Cardiomyopathy subtypes and AF were identified from *International Classification of Diseases, Tenth Revision, Clinical Modification* (*ICD‐10‐CM*) codes in patient EMRs: I42.xx (cardiomyopathy), I42.1 and I42.2 (hypertrophic cardiomyopathy), I42.5 (restrictive cardiomyopathy), I42.0 (dilated cardiomyopathy), I48.xx (atrial fibrillation and flutter). Correspondingly, AF was an exclusion criterion in the matched controls. This study is reported as per the STROBE (*Strengthening the Reporting of Observational Studies in Epidemiology*) guidelines. As a federated network, research studies using the TriNetX research network do not require ethical approvals because no patient‐identifiable information is received. The TriNetX database internally performs extensive data quality assessment with every refresh based on conformance, completeness, and plausibility.^5^

### Data Collection

The TriNetX network was searched on January 17, 2021. The cardiomyopathy and AF cohorts were aged ≥18 years, with a diagnosis of cardiomyopathy at least 1 year before to allow for 1‐year follow‐up. Controls were aged ≥18 years with a diagnosis of cardiomyopathy at least 1‐year before and no history of AF. At the time of the search, 49 participating health care organizations had data available for patients who met the study inclusion criteria.

### Statistical Analysis

All statistical analyses were completed on the TriNetX online platform. Baseline characteristics were compared using χ^2^ tests for categorical variables and independent‐sample *t* tests for continuous variables. Propensity‐score matching (PSM) was used to control for differences in the AF and control cohorts, and/or known risk factors for cardiovascular disease and all‐cause mortality. Patients with cardiomyopathy and AF were 1:1 propensity‐score matched to patients with cardiomyopathy and no record of AF using logistic regression for age at cardiomyopathy diagnosis, sex, race, hypertension, ischemic heart disease, heart failure, cerebrovascular disease, diabetes, chronic kidney disease, cardiovascular procedures (eg, cardiography, echocardiography, cardiac catheterization, cardiac devices, electrophysiological procedures), and cardiovascular medications (eg, β‐blockers, antiarrhythmics, diuretics, antilipemic agents, antianginals, calcium channel blockers, angiotensin‐converting enzyme inhibitors). These variables were chosen because they are established risk factors for cardiovascular disease and/or mortality or were significantly different between the 2 cohorts. The TriNetX platform uses greedy nearest‐neighbor matching, with a caliper of 0.1 pooled standard deviations.

Following PSM, logistic regressions produced odds ratios (ORs) with 95% CIs for 1‐year (from cardiomyopathy diagnosis) outcomes (*ICD‐10‐CM* codes); all‐cause mortality (D; deceased), hospitalization (1013659; hospital inpatient services), incident heart failure (I50), and incident stroke (I63; cerebral infarction), comparing patients with and without AF for each cardiomyopathy subtype. Logistic regression was also used to produce ORs and 95% CIs to investigate the associations with all‐cause mortality comparing patients with AF (I48) who received catheter ablation (Z98.89; has had cardiac radiofrequency catheter ablation) to propensity‐score matched patients who did not receive catheter ablation for each cardiomyopathy subtype. Statistical significance was set at *P*<0.05.

## Results

### Patient Characteristics

In total, 634 885 patients from 49 health care organizations had a diagnosis of cardiomyopathy between 2002 and 2020 with at least 1 year of follow‐up. Of the total patients with cardiomyopathy, there were 14 675 (2.3%) patients with hypertrophic, 90 117 (7.0%) patients with restrictive, and 37 685 (5.9%) patients with dilated cardiomyopathy with concomitant AF. Compared with controls, all cardiomyopathy subtypes with AF were older, had a lower proportion of women, had a higher proportion of people identified as White, and a higher proportion of patients with cardiovascular comorbidities, history of cardiovascular procedures, and cardiovascular medications. These variables were included in subsequent PSM analyses (Tables S1–S3).

Following 1:1 PSM, there were 27 460 patients with hypertrophic, 146 512 patients with restrictive, and 58 676 patients with dilated cardiomyopathy included in the outcome analyses (Table). Although some characteristics remained *statistically* different, all cohorts were deemed to be well balanced on age, sex, race, health conditions, cardiovascular procedures, and cardiovascular medications (Tables S1–S3).

### Clinical Outcomes

Following PSM, 1‐year all‐cause mortality was 6.0% in patients with hypertrophic cardiomyopathy and AF, and 4.9% in the matched controls without AF (*P*<0.0001) (OR, 1.26 [95% CI, 1.13–1.40]). All‐cause mortality was 7.1% in patients with dilated cardiomyopathy and AF, and 5.3% in the matched controls without AF (*P*<0.0001) (OR, 1.36 [95% CI, 1.27–1.46]). All‐cause mortality was 6.9% in patients with restrictive cardiomyopathy and AF, and 6.8% in the matched controls without AF (*P*=0.378) (OR, 0.98 [95% CI, 0.94–1.02].

Following PSM, odds of hospitalization, incident HF, and incident stroke at 1 year from cardiomyopathy diagnosis were significantly associated with concomitant AF across all cardiomyopathy subtypes (Table). Catheter ablation was associated with significantly lower odds of all‐cause mortality at 12‐months across all cardiomyopathy subtypes, compared with PSM controls who did not receive ablation (Table).

## Discussion

Collectively, this retrospective analysis represents the largest follow‐up data set of its kind for patients with cardiomyopathy and AF. First, the findings of the present study show that concomitant AF with a diagnosis of hypertrophic and dilated cardiomyopathy, but not restrictive cardiomyopathy, was associated with significantly higher odds of 1‐year all‐cause mortality relative to patients with cardiomyopathy without AF. Second, odds of hospitalization, incident HF, and incident stroke were significantly higher in cardiomyopathy patients with AF compared with patients without AF. Third, among the patients with AF, catheter ablation was associated with significantly lower odds of all‐cause mortality across all cardiomyopathy subtypes, compared with patients with AF but without ablation.

In this EMR network cohort study using data from 69.8 million individuals, 634 885 had a diagnosis of cardiomyopathy, of which 62 281 (9.8%) had hypertrophic (14 675 [23.6%] with concomitant AF), 212 201 (33.4%) had restrictive (90 117 [42.5%] with concomitant AF), and 84 784 (13.4%) had dilated (37 685 [44.4%] with concomitant AF). There is a scarcity of evidence for the prevalence of AF in patients with cardiomyopathy subtypes, especially nonhypertrophic cardiomyopathies. Some previous work has shown an 18% AF prevalence in 3 673 patients with hypertrophic cardiomyopathy,^6^ 15% AF prevalence in 248 patients with arrhythmogenic right ventricular cardiomyopathy,^7^ and 33% AF prevalence in 156 patients with familial dilated cardiomyopathy, which was not significantly different compared with nonfamilial dilated cardiomyopathy (28%, n=289; *P*=0.24).^8^ However, these samples are substantially smaller than the present study, and the real‐world prevalence of AF in cardiomyopathy subtypes has been largely unknown.

The findings of the present study suggest that AF is associated with increased odds of mortality for hypertrophic and dilated, but not restrictive, cardiomyopathy. Rationale for the observed lack of higher odds of mortality for patients with restrictive cardiomyopathy and concomitant AF is unknown. Particularly given the comparable age group and prevalence of comorbidities compared with hypertrophic and dilated cardiomyopathy cohorts in this study. It is perhaps plausible that the patients with restrictive cardiomyopathy had more advanced cardiomyopathy substrate, which limits the detrimental impact of concomitant AF. It is interesting, however, that catheter ablation is associated with lower mortality in this cohort. Further mechanistic work here is therefore warranted.

Most research to date investigating the prevalence and impact of AF in patients with cardiomyopathy has focused on hypertrophic cardiomyopathy.^9^ AF has been deemed an important risk factor for overall mortality in one prospective study (n=509) with more than a 3‐fold increased risk of death in patients with hypertrophic cardiomyopathy compared with those without AF.^10^ In a larger sample of 3 673 patients with hypertrophic cardiomyopathy, of which 18% were diagnosed with AF, Siontis et al reported that AF was associated with an increased risk of all‐cause mortality (hazard ratio [HR], 1.76).^6^ In the present study of 27 460 patients with hypertrophic cardiomyopathy, AF was associated with 26% increased odds of all‐cause mortality.

Previous work has identified variable impacts of AF on adverse health outcomes relative to the subtype of cardiac disease being studied. For example, Olson et al found AF was associated with a greater relative increase in the risk of adverse outcomes in patients with preserved ejection fraction (HR, 1.72) compared with patients with reduced ejection fraction (HR, 1.29).^11^ In contrast, in the present study, AF was not associated with significantly increased odds of mortality in patients with restrictive cardiomyopathy (OR, 1.01 [95% CI, 0.97–1.04]), but mortality risk was higher in patients with dilated cardiomyopathy (OR, 1.09 [95% CI, 1.03–1.15]). Data from the Heart Muscle Disease Registry of Trieste^12^ highlighted the differing impact of baseline and incident AF on outcomes in 539 patients with dilated cardiomyopathy, where there was no association with baseline AF and mortality but a significant association between new‐onset AF and increased mortality (HR, 3.67).

Management and treatment of AF in patients with hypertrophic cardiomyopathy presents a key component of the American Heart Association and American College of Cardiology guidelines.^13^ In addition to mortality, AF and cardiomyopathies have been associated with higher risk of cardiovascular events and disease progression. In a Japanese cohort study of 20 000 patients with AF, both dilated and hypertrophic cardiomyopathy were the strongest risk factors independently associated with an increased risk of thromboembolic events.^14^ Indeed, in the present study, AF was associated with higher odds of hospitalization, incident HF, and incident stroke across hypertrophic, restrictive, and dilated cardiomyopathies (Table).

Although less well researched, restrictive cardiomyopathy is an important subgroup of patients. In the present study with 146 512 patients with restrictive cardiomyopathy, AF was associated with 43%, 88%, and 79% increased odds for hospitalization, incident HF, and incident stroke, respectively, but not all‐cause mortality. It is conceivable that a longer observation period of >12 months would have allowed for these important clinical events to translate into lower mortality too. Further research into the treatment and management of this population is therefore warranted.

Collectively, the prevalence of AF in patients with cardiac disease seems to be strongly related to the severity of HF, with increasing severity associated with increased AF prevalence. Even a diagnosis of paroxysmal AF has been associated with a greater degree of structural left atrial remodeling and global myopathy, which suggests a more severe cardiomyopathy phenotype.^15^ This likely explains the increased risk of adverse outcomes and new‐onset cardiovascular conditions seen with concomitant AF across all cardiomyopathy subtypes in the present study.

Catheter ablation for AF in patients with HF has been associated with improved cardiac function, symptoms, exercise capacity, and quality of life, and more recently, a significantly lower rate of a composite of all‐cause mortality or hospitalization compared with medical therapy.^16^ Similar observations have also been shown for improvements in left ventricular ejection fraction, restoring sinus rhythm, and freedom from AF following ablation in patients with HF.^16^, ^17^, ^18^ In the present study, an EMR of catheter ablation for AF was associated with 29%, 43%, and 19% lower odds of mortality in patients with hypertrophic, restrictive, and dilated cardiomyopathy, respectively. This was compared with propensity‐score matched patients with AF and cardiomyopathy, but without ablation. These findings are congruent with previous work; a retrospective analysis of patients with persistent AF and concomitant tachycardia‐induced cardiomyopathy had more favorable outcomes following catheter ablation compared with those without cardiomyopathy.^19^ Albeit in a small sample (n=45 with cardiomyopathy), survival at 3 years following ablation was higher in the cardiomyopathy cohort (69%) compared with the noncardiomyopathy cohort (42%). In long‐term follow‐up studies, results comparable to the present study have been documented, although in a hypertrophic cardiomyopathy population only. Among 566 patients with primary hypertrophic cardiomyopathy, Higuchi et al^20^ followed those who were managed for AF with catheter ablation (n=34) and those without (n=60). During a mean follow‐up of 5.8 years, the incidence of clinical events (cardiomyopathy‐related death, hospitalization, or incident thromboembolic stroke) was significantly lower in patients who received ablation compared with nonablation. In a Cox multivariate analysis, catheter ablation therapy was the only independent predictor of incident clinical events. It has been suggested that atrial remodeling following catheter ablation may explain the beneficial impact in patients with cardiomyopathy. To further explain this, Sugumar et al^21^ demonstrated reverse electrical and structural atrial recovery simultaneous with recovery of left ventricular systolic function 2 years after AF ablation in patients with AF‐mediated cardiomyopathy. This may partially explain the long‐term success of catheter ablation in patients with AF and concomittant cardiomyopathy.

### Limitations

Several limitations are noteworthy. First, the data were collected from health care organization EMR databases, and some health conditions may be underreported. Recording of *ICD‐10‐CM* codes in administrative data sets may vary by factors such as age, number of comorbidities, severity of illness, length of hospitalization, and whether in‐hospital death occurred.^22^ We could also not determine the influence of attending different health care organizations because of data privacy restrictions. In addition, outcomes that occurred outside of the TriNetX network are not well captured. It was not possible to investigate clinical outcomes of patients with AF and arrhythmogenic right ventricular cardiomyopathy, given that the *ICD‐10‐CM* code I42.8, which includes arrhythmogenic right ventricular cardiomyopathy, is defined as “other cardiomyopathies” and is therefore nonspecific. Second, the data were largely from multiple health care organizations in the United States but may not be representative of the wider population, thus the generalizability of the results beyond this cohort is unclear. Third, data over longer follow‐up time periods would be valuable, particularly for mortality and cardiovascular disease outcomes. Fourth, future work including more detailed investigation of cardiac function (eg, left ventricular ejection fraction) and AF subtypes is encouraged, which would likely be more feasible in smaller, prospective studies. Finally, it is possible that selection bias affected the improved outcome in patients with AF who recieved ablation. For example, patients subjected to ablation might be healthier, have a higher socioeconomic standing, and receive better quality care, and therefore randomized controlled trials would be needed to investigate the causal effects of ablation therapy in this cohort. Similarly, residual confounding may have impacted our results, including lifestyle factors and socioeconomic status, which were not available from EMRs. This may be particularly true for the catheter ablation outcomes. In the CASTLE AF (Catheter Ablation Versus Standard Conventional Therapy in Patients With Left Ventricular Dysfunction and Atrial Fibrillation) trial, the only positive randomized controlled trial to date showing mortality benefit with catheter ablation in heart failure, the mortality curves only started to diverge in the third year.^16^ Therefore our promising results for AF ablation in patient with cardiomyopathy at 12 months may be impacted by residual confounding.

## Conclusions

Using a global federated health research network, we found AF may be highly prevalent in patients with cardiomyopathy and associated with worsened prognosis. Concomittant AF with hypertrophic and dilated, but not restrictive cardiomyopathy associated with significantly higher odds of mortality. Though, all cardiomyopathy subtypes were associated with significantly higher odds of hospitalization, incident HF, and incident stroke when present with AF. Catheter ablation was associated with significantly lower odds of mortality across all cardiomyopathy subtypes, when compared to matched patients who did not recieve ablation procedures. The findings of the present study suggest that patients with cardiomyopathy and AF associate with worsened prognosis, and subsequent prospective research is needed to determine the usefulness of screening and treating AF in this population. This is particularly true for cardiomyopathies other than hypertrophic cardiomyopathy, which seem underresearched.

## Sources of Funding

None.

## Disclosures

Dr Buckley has received funding from Bristol‐Myers Squibb/Pfizer. Dr Harrison has received funding from Bristol‐Myers Squibb. E. Fazio‐Eynullayeva and P. Underhill are employees of TriNetX Inc. Dr Gupta has received institutional research grants from Boston Scientific, Medtronic, and Biosense Webster, and personal advisory fees from Boehringer Ingelheim, Boston Scientific and Abbott. Dr Lip is a consultant for Bayer/Janssen, Bristol‐Myers Squibb/Pfizer, Medtronic, Boehringer Ingelheim, Novartis, Verseon, and Daiichi‐Sankyo, and speaker for Bayer, Bristol‐Myers Squibb/Pfizer, Medtronic, Boehringer Ingelheim, and Daiichi‐Sankyo. No fees are directly received personally. The remaining authors have no disclosures to report.

**Table 1:** One‐Year Major Adverse Events/Conditions from Cardiomyopathy Diagnosis Comparing Patients With Cardiomyopathy and AF to Propensity‐Matched Patients With Cardiomyopathy Only

Major adverse events/conditions	No. of participants^†^	Odds ratio	95% CI	*P* value
Hypertrophic cardiomyopathy	27 460			
Mortality		1.26	1.13–1.40	<0.0001
Hospitalization		1.45	1.37–1.53	<0.0001
Incident HF		2.87	2.61–3.16	<0.0001
Incident stroke		1.77	1.50–2.10	<0.0001
Mortality following catheter ablation*	10 212	0.71	0.60–0.83	<0.0001
Restrictive cardiomyopathy	146 512			
Mortality		0.98	0.94–1.02	0.378
Hospitalization		1.43	1.39–1.46	<0.0001
Incident HF		1.88	1.81–1.95	<0.0001
Incident stroke		1.79	1.66–1.94	<0.0001
Mortality following catheter ablation*	56 010	0.57	0.53–0.61	<0.0001
Dilated cardiomyopathy	58 676			
Mortality		1.36	1.27–1.46	<0.0001
Hospitalization		1.60	1.53–1.66	<0.0001
Incident HF		1.50	1.40–1.62	<0.0001
Incident stroke		1.55	1.36–1.76	<0.0001
Mortality following catheter ablation*	22 040	0.81	0.74–0.89	<0.0001

AF indicates atrial fibrillation; and HF, heart failure.

* Logistic regression analyses comparing patients with cardiomyopathy and AF who received catheter ablation with matched patients who have not received ablation.

^†^ Sample size: cohort with cardiomyopathy and AF plus cohort with cardiomyopathy without AF.

## Supporting information

Tables S1–S3
