# Supplementary material for: Atrial Fibrillation in Patients With Cardiomyopathy: Prevalence and Clinical Outcomes From Real‐World Data
Source: J Am Heart Assoc. 2021 Nov 15;10(23):e021970. doi: 10.1161/JAHA.121.021970 (PMC9075382; doi:10.1161/JAHA.121.021970)
Supplement: Supplementary file 1 — Tables S1–S3 [file JAH3-10-e021970-s001.pdf]

SUPPLEMENTAL MATERIAL

Table S1. Baseline characteristics %(*n*)* of the HCM populations with and without AF before and after propensity score matching.

	**Initial populations**			**Propensity score matched populations**			
	HCM without AF (n=47,606)	HCM with AF (n=14,675)	*P*-value	HCM without AF (n=13,730)	HCM with AF (n=13,730)	*P*-value	SMD
Age (years) at diagnoses; mean (SD)	52.6 (20.0)	65.3 (14.6)	<0.0001	64.5 (14.5)	64.7 (14.5)	0.153	0.017
Female	52.3 (24,916)	46.0 (6,755)	<0.0001	46.7 (6,415)	47.0 (6,447)	0.699	0.005
Male	47.7 (22,686)	53.9 (7,916)	<0.0001	53.3 (7,312)	53.0 (7,280)	0.699	0.005
Ethnicity							
White	56.1 (26,704)	72.2 (10,597)	<0.0001	71.6 (9,837)	70.9 (9,736)	0.178	0.016
Black or African American	31.5 (15,018)	18.5 (2,709)	<0.0001	18.8 (2,583)	19.4 (2,664)	0.214	0.015
Asian	2.2 (1,031)	1.6 (240)	<0.0001	1.6 (219)	1.7 (237)	0.395	0.010
Unknown	9.7 (4,633)	7.4 (1,085)	<0.0001	7.7 (1,052)	7.6 (1,050)	0.964	0.001
Comorbidities							
Hypertensive diseases	33.3 (15,836)	44.6 (6,552)	<0.0001	41.0 (5,630)	42.6 (5,849)	0.007	0.032
Ischaemic heart diseases	9.4 (4,461)	22.6 (3,314)	<0.0001	18.8 (2,577)	19.8 (2,714)	0.036	0.025
Heart failure	6.9 (3,272)	22.6 (3,313)	<0.0001	16.6 (2,274)	18.0 (2,473)	0.001	0.038
Diabetes Mellitus	13.6 (6,451)	17.0 (2,498)	<0.0001	15.4 (2,119)	16.3 (2,235)	0.055	0.023
Chronic Kidney Disease	6.0 (2,865)	12.4 (1,821)	<0.0001	9.8 (1,351)	10.7 (1,464)	0.025	0.027
Cerebrovascular diseases	4.2 (1,991)	8.8 (1,295)	<0.0001	7.3 (1,001)	7.8 (1,075)	0.091	0.020
Cardiovascular care							
Cardiovascular Procedures[b]	27.7 (13,189)	43.6 (6,396)	<0.0001	40.9 (5,621)	40.9 (5,618)	0.971	0.000
Cardiovascular Medications[c]	42.6 (20,274)	56.0 (8,217)	<0.0001	53.4 (7,331)	54.0 (7418)	0.292	0.013

*Values are % (n) unless otherwise stated. Baseline characteristics were compared using a chi-squared test for categorical variables and an independent-sample t-test for continuous variables. [a]Data are taken from structured fields in the electronic medical record systems of the participating healthcare organizations, therefore, there may be regional or country-specific differences in how race categories are defined. [b]Cardiovascular procedures include cardiography, echocardiography, catheterization, cardiac devices, electrophysiological procedures. [c]Cardiovascular medications include beta-blockers, antiarrhythmics, diuretics, lipid lowering agents, antianginals, calcium channel blockers, ACE inhibitors. AF; atrial fibrillation, HCM; hypertrophic cardiomyopathy, SD; standard deviation, SMD; standardised mean difference. The cardiomyopathy and AF cohorts were distributed between the four large Census Bureau designated regions of the United States as follows: 18% in the Northeast, 17% in the Midwest, 45% in the South, 10% in the West, and 10% were unknown. The control (non-AF) cohort was distributed as follows: 17% in the Northeast, 20% in the Midwest, 43% in the South, 9% in the West, 1% non-United States, and 10% were unknown.

Table S2. Baseline characteristics %(*n*)* of the restrictive cardiomyopathy populations with and without AF before and after propensity score matching.

	Initial populations			Propensity score matched populations			
	Restrictive CM without AF (n=122,084)	Restrictive CM with AF (n=90,117)	*P*-value	Restrictive CM without AF (n=73,256)	Restrictive CM with AF (n=73,256)	*P*-value	SMD
Age (years) at diagnoses; mean (SD)	55.8 (18.2)	66.5 (14.1)	<0.0001	63.8 (13.9)	63.8 (13.9)	0.856	0.001
Female	43.2 (52,692)	36.2 (26,484)	<0.0001	35.3 (25,861)	36.2 (26,484)	0.001	0.018
Male	56.8 (69,381)	63.8 (46,764)	<0.0001	64.7 (47,387)	63.8 (46,764)	0.001	0.018
Ethnicity							
White	60.4 (73,715)	69.4 (50,873)	<0.0001	68.8 (50,428)	69.4 (50,873)	0.012	0.013
Black or African American	23.4 (28,610)	18.8 (13,795)	<0.0001	19.7 (14,427)	18.8 (13,795)	<0.001	0.022
Asian	1.3 (1,637)	1.1 (785)	<0.0001	1.0 (707)	1.1 (785)	0.042	0.011
Unknown	14.5 (17,662)	10.3 (7,578)	<0.0001	10.2 (7,490)	10.3 (7,578)	0.449	0.004
Comorbidities							
Hypertensive diseases	33.4 (40,758)	39.3 (28,769)	<0.0001	38.4 (28,129)	39.3 (28,769)	0.001	0.018
Heart failure	24.5 (29,952)	31.3 (22,959)	<0.0001	30.8 (22,569)	31.3 (22,959)	0.028	0.012
Ischaemic heart diseases	22.0 (26,799)	27.7 (20,324)	<0.0001	27.0 (19,765)	27.7 (20,324)	0.001	0.017
Diabetes Mellitus	16.2 (19,789)	19.0 (13,900)	<0.0001	18.3 (13,411)	19.0 (13,900)	0.001	0.017
Chronic Kidney Disease	9.0 (11,016)	11.3 (8,314)	<0.0001	10.8 (7,879)	11.3 (8,314)	<0.001	0.019
Cerebrovascular diseases	5.3 (6,416)	7.0 (5,132)	<0.0001	6.5 (4,774)	7.0 (5,132)	<0.001	0.019

Cardiovascular care							
Cardiovascular Procedures[b]	37.6 (45,936)	41.0 (30,055)	<0.0001	40.6 (29,731)	41.0 (30,055)	0.085	0.009
Cardiovascular Medications[c]	39.4 (48,085)	45.1 (33,005)	<0.0001	44.9 (32,887)	45.1 (33,005)	0.535	0.003

*Values are % (n) unless otherwise stated. Baseline characteristics were compared using a chi-squared test for categorical variables and an independent-sample t-test for continuous variables. [a]Data are taken from structured fields in the electronic medical record systems of the participating healthcare organizations, therefore, there may be regional or country-specific differences in how race categories are defined. [b]Cardiovascular procedures include cardiography, echocardiography, catheterization, cardiac devices, electrophysiological procedures. [c]Cardiovascular medications include beta-blockers, antiarrhythmics, diuretics, lipid lowering agents, antianginals, calcium channel blockers, ACE inhibitors. AF; atrial fibrillation, CM; cardiomyopathy, SD; standard deviation, SMD; standardised mean difference. The cardiomyopathy and AF cohorts were distributed between the four large Census Bureau designated regions of the United States as follows: 18% in the Northeast, 17% in the Midwest, 45% in the South, 10% in the West, and 10% were unknown. The control (non-AF) cohort was distributed as follows: 17% in the Northeast, 20% in the Midwest, 43% in the South, 9% in the West, 1% non-United States, and 10% were unknown.

Table S3. Baseline characteristics %(*n*)* of the dilated cardiomyopathy populations with and without AF before and after propensity score matching.

	Initial populations			Propensity score matched populations			
	Dilated CM without AF (n=47,099)	Dilated CM with AF (n=37,685)	*P*-value	Dilated CM without AF (n=29,338)	Dilated CM with AF (n=29,338)	*P*-value	SMD
Age (years) at diagnoses; mean (SD)	57.0 (17.5)	66.5 (13.7)	<0.0001	63.7 (13.5)	63.8 (13.6)	0.444	0.006
Female	40.7 (19,146)	30.5 (11,484)	<0.0001	33.8 (9,910)	34.1 (9,993)	0.469	0.006
Male	59.3 (27,921)	69.5 (26,193)	<0.0001	66.2 (19,419)	65.9 (19,337)	0.475	0.006
Ethnicity							
White	58.7 (27,668)	71.8 (27,064)	<0.0001	67.2 (19,723)	67.7 (19,857)	0.238	0.010
Black or African American	24.0 (11,303)	17.8 (6,704)	<0.0001	21.5 (6,298)	20.4 (5,977)	0.001	0.027
Asian	5.5 (2,576)	1.4 (532)	<0.0001	1.2 (345)	1.8 (530)	<0.001	0.052
Unknown	11.3 (5,333)	8.6 (3,259)	<0.0001	9.8 (2,868)	9.8 (2,864)	0.956	0.000
Comorbidities							
Hypertensive diseases	38.0 (17,881)	51.7 (19,498)	<0.0001	45.5 (13,363)	46.4 (13,614)	0.038	0.017
Heart failure	34.9 (16,419)	50.2 (18,916)	<0.0001	42.9 (12,599)	43.1 (12,656)	0.635	0.004
Ischaemic heart diseases	22.1 (10,403)	33.9 (12,763)	<0.0001	27.9 (8,197)	28.6 (8,380)	0.093	0.014
Diabetes Mellitus	17.2 (8,093)	23.0 (8,664)	<0.0001	20.5 (6,013)	21.0 (6,151)	0.160	0.012
Chronic Kidney Disease	11.3 (5,310)	19.7 (7,411)	<0.0001	14.4 (4,236)	15.0 (4,415)	0.037	0.017
Cerebrovascular diseases	4.8 (2,258)	9.0 (3,405)	<0.0001	6.3 (1,858)	6.7 (1,965)	0.073	0.015
Cardiovascular care							

Cardiovascular Procedures[b]	42.0 (19,791)	53.4 (20,139)	<0.0001	49.0 (14,366)	48.9 (14,345)	0.862	0.001
Cardiovascular Medications[c]	56.5 (26,628)	65.5 (24,665)	<0.0001	60.3 (17,687)	61.4 (18,012)	0.006	0.023

*Values are % (n) unless otherwise stated. Baseline characteristics were compared using a chi-squared test for categorical variables and an independent-sample t-test for continuous variables. [a]Data are taken from structured fields in the electronic medical record systems of the participating healthcare organizations, therefore, there may be regional or country-specific differences in how race categories are defined. [b]Cardiovascular procedures include cardiography, echocardiography, catheterization, cardiac devices, electrophysiological procedures. [c]Cardiovascular medications include beta-blockers, antiarrhythmics, diuretics, lipid lowering agents, antianginals, calcium channel blockers, ACE inhibitors. AF; atrial fibrillation, CM; cardiomyopathy, SD; standard deviation, SMD; standardised mean difference. The cardiomyopathy and AF cohorts were distributed between the four large Census Bureau designated regions of the United States as follows: 18% in the Northeast, 17% in the Midwest, 45% in the South, 10% in the West, and 10% were unknown. The control (non-AF) cohort was distributed as follows: 17% in the Northeast, 20% in the Midwest, 43% in the South, 9% in the West, 1% non-United States, and 10% were unknown.
